# Visions of recovery: a cross-diagnostic examination of eating disorder pro-recovery communities on TikTok

**DOI:** 10.1186/s40337-023-00827-7

**Published:** 2023-07-03

**Authors:** Amanda K. Greene, Hannah N. Norling, Lisa M. Brownstone, Elana K. Maloul, Caity Roe, Sarah Moody

**Affiliations:** 1 Center for Bioethics and Social Sciences in Medicine, University of Michigan Medical School, 2800 Plymouth Road, Bldg. 14, G016, Ann Arbor, MI 48109 USA; 2 Department of Counseling Psychology, Morgridge College of Education, University of Denver, 1999 East Evans Avenue, Denver, CO 80208-1700 USA; 3 Department of English Language and Literature, University of Michigan, 435 South State Street, Ann Arbor, MI 48104 USA

**Keywords:** Eating disorders, Pro-recovery, Social media, TikTok, Diet culture

## Abstract

Individuals seeking support or inspiration for eating disorder recovery may turn to pro-recovery content on social media sites such as TikTok. While research has thus far treated pro-recovery social media as a fairly homogeneous space, many pro-recovery hashtags single out particular eating disorder diagnoses. This exploratory study used codebook thematic analysis of 241 popular pro-recovery videos on TikTok to compare the presentation of eating disorders and eating disorder recovery across five different diagnosis-specific hashtags: #anarecovery, #arfidrecovery, #bedrecovery, #miarecovery, and #orthorexiarecovery. These hashtags refer to the following eating disorder diagnoses respectively: anorexia nervosa, avoidant restrictive food intake disorder, binge eating disorder, bulimia nervosa, and orthorexia nervosa. Our analysis generated the following qualitative themes across the entire dataset: (1) *centrality of food to eating disorders and recovery*, (2) *what eating disorders look and feel like*, (3) *recovery as a process*, (4) *getting and giving help*, and (5) *negotiating diet culture in recovery*. To supplement our qualitative findings and facilitate cross-diagnostic comparisons, we also conducted one-way ANOVAs and chi-square tests to probe for statistically significant differences in audience engagement and code prevalence across the different hashtags. Our results indicate that there are clear differences in how recovery is envisioned on TikTok based on which diagnostic hashtags are employed. Such variations in how different eating disorders are imagined on popular social media demand further investigation and clinical consideration.

**Supplementary Information:**

The online version contains supplementary material available at 10.1186/s40337-023-00827-7.

## Abstract (plain language summary)

Hashtags related to eating disorder recovery on TikTok often name particular eating disorder diagnoses. This study compared five of these diagnosis-specific hashtags: #anarecovery, #arfidrecovery, #bedrecovery, #miarecovery, and #orthorexiarecovery. We found some similarities among these hashtags such as the centrality of food and eating in the posts and the emphasis on recovery as a process. However, we also found significant differences between the hashtags. For example, while diet culture promotion was a key aspect of many #bedrecovery posts, #orthorexiarecovery posts tended to focus instead on critiques of diet culture. Levels of user engagement also varied across the five hashtags. Notably, #anarecovery posts received the most likes. This study points to the existence of subcommunities within pro-recovery social media and suggests that way eating disorder recovery is portrayed online differs across diagnostic labels.

**Supplementary Information:**

The online version contains supplementary material available at 10.1186/s40337-023-00827-7.

## Introduction

There is a growing body of evidence that user engagement with social media promoting dieting, eating disorders or the thin ideal is associated with disordered eating symptomatology, body image difficulties, and other negative mental health outcomes (such as anxiety and depression) [1–3]. More specifically, work on pro-eating disorder (pro-ED) online communities has demonstrated the harms of online spaces that applaud, affirm, and share information supporting eating disordered behaviors and identities [4, 5]. Responding to the proliferation of pro-ED content, pro-eating disorder recovery (pro-recovery) communities have now become highly present on various social media platforms. Some recent scholarship has begun to investigate these pro-recovery spaces across established social media platforms. However, with the rapid popularization of TikTok in the last few years, more research is needed to understand how eating disorder content manifests on this important newcomer to the social media ecosystem. Additionally, previous research has tended to treat pro-recovery spaces as fairly homogenous in spite of the fact that many pro-recovery hashtags name specific eating disorder diagnoses. The current study examines pro-recovery TikTok by describing and comparing popular videos across five diagnostic hashtags:#anarecovery, #arfidrecovery, #bedrecovery, #miarecovery, and #orthorexiarecovery (referring respectively to anorexia nervosa (AN), avoidant restrictive food intake disorder (ARFID), binge eating disorder (BED), bulimia nervosa (BN), and orthorexia nervosa (ON)) [6, 7]. A better understanding of the differences between these recovery-oriented subcommunities can help clinicians be more attuned to the heterogeneity of how specific eating disorders are represented online as well as the ways these digital cultures intersect with in-person presentations and shape expectations for recovery.

### TikTok and mental health

Since its worldwide debut in 2016, TikTok has grown quickly in popularity and become an important hub for younger demographics to consume and share content. According to recent studies, TikTok has roughly 850 million downloads, 689 million active users worldwide, and over a billion new videos each day [8]. Of those active users, 60% are between 16 and 24 years of age [9]. TikTok posts take the form of short videos, ranging from just a few seconds to three minutes. Similar to other platforms, users can connect on the site by following particular accounts, as well as through @ mentions and hashtags. TikTok is also notorious for its black-boxed algorithmic recommendations in the form of the “For You” Page, designed to extend users’ time on the platform by providing targeted content based on their previous watch history.

Among the myriad content available on TikTok, mental health is one prevalent topic that receives high levels of engagement [10]. Audience interaction with mental health content here often appears positive, with users offering social support, personal narratives, and advice to the original content creator [11]. Videos that utilize the #mentalhealth hashtag often fit into subcategories of helpful and affirming content, including informational, anecdotal, accomplishment sharing, and humor [9]. Such content and communities may benefit users by providing peer support for individuals with shared experiences living with mental health concerns, even creating a positive atmosphere to reduce stigma and increase individuals’ ability to advocate for themselves [9].

Conversely, the ability for virtually anyone to make mental health content has presented unique challenges. Among these is the spread of mis/information about mental health and a variety of mental illnesses [10, 12, 13]. Research has suggested this mis/information spread on sites like TikTok has coincided with a rise in young social media users identifying with a variety of mental health concerns, including less prevalent disorders like Dissociative Identity Disorder and Tourettes [14–17]. Even more noxious impacts of social media use—including psychic distress, anxiety, depression, body image disturbance, self-injurious behavior, and suicidality—have also been found in a number of studies [3, 18–20].

### Eating disorders and social media

Pro-ED blogs and websites have long existed as spaces for individuals to share their eating disorder experiences and identities with like-minded individuals. There is a substantial body of scholarship documenting the existence of many pro-ED communities online that promote eating disorders as a lifestyle choice as opposed to a mental illness [21–24]. “Thinspiration,” which includes photos or videos of thin bodies that provide aspirational imagery for viewers looking to achieve the same level of thinness, is widely circulated within these spaces [25]. Evidence suggests that consumption of “thinspiration” and pro-ED content is damaging to users, as it can be associated with higher body dissatisfaction [26], higher likelihood of disordered eating behaviors, and poorer quality of life [27, 28]. In response to the studies indicating that these online spaces are damaging, social media sites such as TikTok [29, 30] and Instagram [31] have attempted to remove both hashtags and content promoting eating disorder behaviors on their platform. Nonetheless, pro-ED content is still easy to find. Clever misspellings of hashtags like #edsheeran and #eatingdis0rder allow eating disorder content to escape censorship and endure on TikTok [29, 30].

Although pro-ED spaces are often positioned as dangerous, some literature centers pro-ED online as a place where information sharing, community building and support seeking can happen for individuals who do not wish to recover from their eating disorder [4, 5, 32, 33]. These studies make the case that online pro-ED communities may be some of the only places where individuals who still struggle with their eating disorder can feel a sense of relief from public surveillance and avoid scrutiny. This perception of freedom may allow creators to more authentically express struggles and, ultimately, what they see as triumphs in their eating disorders [34, 35].

Pro-recovery spaces online appear to offer a similar support system in spite of their different emphasis. They can often give individuals who are inclined to recover a place to build community, find recovery motivation and inspiration, engage in accountability, and seek support [36]. However, research also shows that even pro-recovery spaces are not devoid of harmful content because diet culture, triggering imagery, comparisons, and a focus on appearance may also appear there [36]. Differentiating between pro-ED and pro-recovery content can sometimes be difficult as a result of their similarity in content and structure, leading users to find frequent cross-contamination in these spaces [37–40]. Due to the nature of TikTok’s algorithm and how it populates feeds with similar videos for users, it is possible that pro-ED and pro-recovery content is similar enough that they may cross-pollinate, making it especially difficult for users to avoid emotionally triggering posts with content that they were not originally seeking [41].

Research on pro-recovery TikTok has been fairly limited thus far. In one important study, an inductive thematic analysis of the hashtag #EDRecovery on the platform, Herrick et al. (2020) suggested the TikTok pro-recovery community has a wide range of themes that foster individual voices and solidarity. These themes included *eating disorder awareness, inpatient storytime, eating in recovery, transformations,* and *trendy gallows humor*. Herrick and colleagues [39] in their discussion, also highlighted the seemingly unavoidable cross contamination of pro-recovery and pro-ED in the #EDRecovery hashtag. Additional research indicates that “anti-pro-ana” content on TikTok may end up being just as harmful as “pro-ana” content, with creators comparing their levels of “sickness” or even creating tutorials for other users who are wanting to engage in disordered eating behavior [42]. The genuinely supportive content and community building in this pro-recovery space appears to co-exist with other harmful content that may encourage disordered eating [39].

### Differentiating eating disorders based on diagnosis

Further understanding how pro-recovery spaces interact with different diagnostic labels may help us understand a more nuanced pro-recovery landscape that considers the existence of sub-communities within pro-recovery. Diagnostic hashtags are an important and thus far overlooked component of this ecosystem. Differences between these hashtags may be especially consequential given the different societal values and imaginaries that attach to different eating disorder diagnoses. For example, one phenomenological study notes that those with AN who later transitioned to BN or BED experienced more feelings of shame and spoke about the moral superiority of having AN as a diagnosis [43]. This may be attributed to the aspects of control associated with a restrictive eating disorder. In spite of sharing very similar symptoms to BN (fear of weight gain, thinness, and body dissatisfaction), feelings of shame towards binge eating and purging likely suggests that this disorder is lower in the artificial moral hierarchy of eating disorders [44]. Individuals suffering from eating disorders are not the only ones who believe AN is a more moral eating disorder: mass media often portrays BED critically by suggesting that the suffering individual lacks self-control [45]. Comparatively, those who suffer from AN and BN are more often portrayed in media with greater nuance [45]. Scrutiny of BED may also be related to the fact that individuals with BED are more likely to exist in larger bodies, while those with diagnoses of AN or BN tend to exist in smaller [46]. Additionally, research suggests that binging is itself a stigmatized behavior that may contribute to weight stigma regardless of an individual’s weight status [47].

Only a few studies have focused on the representation of specific eating disorder diagnoses online. Analyses of Instagram posts tagged with #orthorexia suggest that this community is largely defined by positive conversation and support around recovery [48]. A more recent mixed-methods study of ON on Twitter emphasized the contested medicalization of this diagnosis in social media conversations, which often compared it to more established diagnoses like BN and AN [49]. In contrast, research on BED and social media has focused on the ways social media use may promote binge eating behaviors, and not on the online representation of BED culture itself [50]. To the authors’ knowledge, no research has yet been conducted on the social media presence of ARFID.

Most of the research on pro-recovery social media has either explicitly or implicitly focused on the diagnoses defined by restriction or compensatory behavior: BN and AN. For example, one review of literature on social media and eating disorders indicated that most studies only used some combination of the words “anorexia,” “bulimia,” “eating disorders,” and “self-esteem” as search terms [51]. Another recent study focused on Instagram only used #EatingDisorderRecovery, #AnorexiaRecovery, #BulimiaRecovery and #RecoveryWarrior in their participant recruitment [36]. The present study addresses the gap in diagnostic-specific considerations of eating disorder pro-recovery on social media. Focus on pro-recovery as an assumed homogenous category may obfuscate more complex illness identities that coalesce around particular diagnostic self-identifications. By examining diagnostic specific communities in pro-recovery, we can further understand how individuals who identify with having certain disorders may be able to access and envision recovery in ways that are specific to their diagnosis or diagnostic self-identification.

## Methods

### Theoretical and methodological orientation

We took an abductive approach to codebook thematic analysis [52] in order to surface new insights from our data while remaining in dialogue with previous literature on pro-recovery TikTok [39]. Given our team’s diversity in terms of discipline and seniority, the codebook structure helped facilitate consistency in the coding while also promoting regular dialogue and researcher reflexivity [53, 54]. We used quantitative data about the codes’ prevalence to supplement our qualitative reflection and to facilitate axial comparisons between the different diagnostic hashtags. These methods are described in more depth below.

### Data collection and organization

Data collection proceeded first by identifying hashtags to target content in pro-recovery communities of the five different disorders that were most commonly represented: AN, ARFID, BED, BN, and ON. These hashtags were #anarecovery, #arfidrecovery, #bedrecovery, #miarecovery, and #orthorexiarecovery. For each disorder we identified one related hashtag that appeared to have the most views based on searching the TikTok platform. This is why, for example, we chose #miarecovery instead of #bulimiarecovery as the hashtag referring to BN. We then used Apify’s TikTok scraper to collect the post URLs and captions along with relevant metadata and information about the poster. This tool collected publicly available posts using one of the hashtags between 1/1/20 and the date of data collection (2/1/22). This process gave us a dataset of 6315 discrete posts, which was then manually cleaned in order to remove posts that had captions or creator bios in a language other than English. We also removed posts from creators self-identifying as minors in order to protect potentially vulnerable individuals. Following cleaning, the dataset was limited to 4640 posts across all five hashtags.

**Fig. 1:**
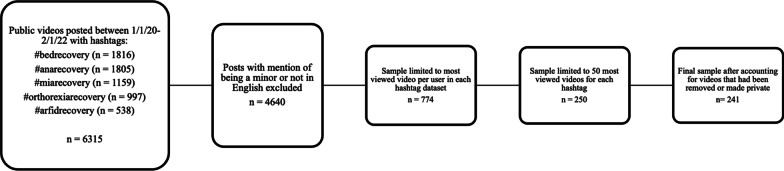
Data collection and organization workflow

To establish a sample of videos for qualitative analysis and avoid our findings being defined by only a couple especially popular content creators, we then limited the dataset to each creator's most-viewed post in each sample of hashtags. Then, the 50 most-viewed posts were selected for analysis. Because some of the videos were removed or made private over the course of our analysis the final sample included 241 videos. See Fig. 1 for a diagram of the data cleaning and organization process.

### Analytic plan

Analysis of the videos began with team bracketing and discussion. All team members filled out a bracketing questionnaire to reflect on their intersecting identities and previous experience, and how positionalities would orient them towards the data. The coding team consisted of 8 individuals who identified as the following: 6/8 cisgender women, 6/8 white, 3/8 queer, 2/8 large-bodied, and 4/8 eating disorder survivors. The team was composed of clinical practitioners, specifically counseling psychology faculty and graduate students, along with a bioethics research analyst and humanities doctoral candidate. We met to collectively discuss our reflections and assumptions, which included that some of the pro-recovery hashtags would be infiltrated with pro-ED content, that weight loss would be more frequently discussed in the #bedrecovery posts, and that content creators in general would be predominantly younger, white women. The team then engaged in a brief literature review focusing on previous content analyses of visual social media content in pro-ED and pro-recovery communities in order to help frame our research questions and develop a shared understanding of codebook development.

We then discussed our research questions and coding priorities. This included deciding that we would not code for body size or the display of particular body parts (e.g., collarbones) that were frequently mentioned in the literature [55, 56]. Team-members came to this decision after expressing concern about reinforcing weight stigma or inappropriately objectifying users. Instead, we decided to prioritize representation of recovery, presence of diet culture, and affective elements of the videos. For the purposes of this project, we drew on the definition of diet culture Jovanovski and Jaeger [57] forwarded through their qualitative survey of individuals in fat activist, feminist, and health professional communities online as “a conflation of weight and health including myths about food and eating, and a moral hierarchy of bodies derived from patriarchal, racist, and capitalist forms of domination.”

Following this initial groundwork, all team members individually coded the same random set of 10 videos. Observations from this first round of pilot coding, in dialogue with prior discussion and understandings of extant literature, were the basis of an initial codebook. All team members then coded another random set of 10 videos using the codebook and met to discuss discrepancies. Through this reflexive discussion, we revised the codebook for clarity and to ensure we were capturing key elements of the videos. Only at this point did we proceed with coding the entire dataset.

For this first round of coding the entire dataset, the videos were not sorted by diagnostic hashtag, but were randomly distributed to team members who watched the videos and coded them together. Although we did not mask the hashtags in the videos and their captions, we chose not to sort them by diagnosis at this stage to avoid developing the codebook around preconceived notions of disorders. Throughout this coding process, the team met weekly to discuss trends and responses to the videos, as well as questions and concerns about the codebook. After coding all the videos in this manner, the team met to revise the codebook one final time by altering specific codes on which team-members had difficulty reaching consensus either by dividing them into different subcodes or combining codes that appeared too fine-grained to be reliably coded. We did another round of pilot coding in pairs to concretize the codebook and descriptions of the codes, in which each pair coded the same two videos per each diagnostic hashtag. See Additional file 1: Appendix A for a list of codes and operational definitions.

**Fig. 2:**
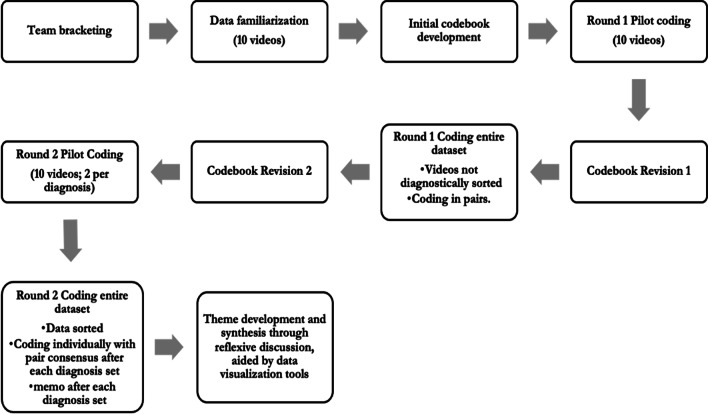
Data analysis workflow

After the team had reached agreement on the revised codebook, we coded the entire dataset again. This time, however, we grouped the posts by their diagnostic hashtag in order to facilitate more concentrated, deep engagement with each particular diagnosis. Pairs were assigned 10 videos per hashtag, which each pair member watched and coded individually before meeting with their partner to establish consensus. Consensus meetings were utilized as an analytic tool to facilitate the development of new insights rather than simple confirmation of reliability. All team members wrote structured memos after coding each set of videos to generate and track insights. These memos and other reflections were discussed at weekly team meetings. For each new hashtag, the same process was followed, though the pairing of team members was rotated to support reflection and idea exchange. After going through a total of 40 videos per hashtag and discussing thematic differences and similarities, the team did one last set of coding to complete remaining videos (with pairs looking at two to three videos per hashtag). We met again after this round was complete to decide if additional coding was necessary and decided that we had reached “information power” [58]. In this meeting, we continued the reflexive analysis and theme synthesis we had begun in the diagnosis specific space, aided by data visualizations of frequencies of different codes across the different hashtags. Tentative themes were developed, which were then iteratively refined through the writing process. Figure 2 presents an overview of this analytic process.

### Integration of quantitative data

After the completion of video coding and the initial development of themes, we integrated quantitative analysis by comparing code frequencies across the diagnostic hashtags. While the primary mode of data analysis was qualitative, this integration of quantitative data was a tool to aid comparison across these five diagnostic hashtags. To examine group differences in continuous dependent variables (i.e., number of plays, number of fans), we completed analysis of variance (ANOVA) with post-hoc Tukey tests. Notably, given that both number of plays and fans were positively skewed, we completed ANOVAs with log(10) transformed data. We present medians in order to best represent the central points of these data given positive skew. For categorical group comparisons, we used chi-square tests to examine whether codes significantly varied between diagnostic groups. To adjust for multiple comparisons we used the Benjamini–Hochberg procedure to assess the statistical significance of overall group differences [59].

We then looked at the significance of individual cells in all of the categorical group comparisons that were significant after applying the Benjamini–Hochberg procedure. Following the procedure outlined by Sharpe [60], we converted adjusted standardized residuals in each cell from a Z-score to a p-value. For these post hoc tests, we set our significance level to *p* = 0.05. We see these quantitative findings as indicative of potential trends that, in dialogue with the qualitative data, suggest differences across diagnostic groups, but require further research and should be interpreted with caution.

## Results

### Overview

For omnibus tests, we report the uncorrected p-values throughout the results. Notably, after the Benjamini–Hochberg procedure, the highest p-value considered statistically significant was *p* = 0.016, and the highest p-value considered marginally significant was *p* = 0.078. See Tables 1 and 2 for information on all tests. Our final dataset included 241 posts. These posts had received a cumulative 61,269,703 plays at the time of data collection (2/1/2022). See Table 1 for descriptive statistics and diagnostic hashtag group differences regarding engagement and demographics. Engagement was measured by number of video plays and number of content creator fans—metadata that was available in the original Apify dataset. An overall marginally significant difference in fans across diagnostic hashtags was observed (*F* (1,236) = 2.63 *p* = 0.035); however, post-hoc Tukey tests did not indicate any significant pairwise differences. On the other hand, we observed an overall significant difference in plays across diagnostic hashtags (*F* (1,236) = 47.48, *p* < 0.001). Post hoc Tukey tests indicated that #anarecovery (Mdn = 233,850) had significantly more plays than all other diagnostic hashtags; #bedrecovery (Mdn = 11,200), #miarecovery (Mdn = 5,689), and #orthorecovery (Mdn = 10,600) had statistically equivalent numbers of plays that were all higher than number of #arfidrecovery plays (Mdn = 1,288) (See Table 1).

**Table 1:** Engagement and demographic data and group differences of posts

	ANA	ARFID	BED	MIA	ORTHO	Total	Significance
Posts (*n*)	50	47	50	45	49	241	
Fans (median, *IQR*)^	8,161^a^ 14,159.3	4,024^a^ 21,937.0	14,600^a^ 57,966.5	7,256^a^ 27,377.5	13,200^a^ 32,324.5	9,113 28,096.5	*F* (1,236) = 2.63 *p* = 0.035
Plays (median, *IQR*)*	233,850^a^ 429,100.0	1,288^c^ 4,637.0	11,200^b^ 29,936.5	5,689^b^ 24,894.5	10,600^b^ 13,929.5	12,800 152,491.0	*F* (1,236) = 47.48 *p* < .001
Female-presenting (*n*, %)*	46 92.0%	33 70.2%*	47 94.0%	42 93.3%	43 87.7%	211 87.6%	χ^2^ (4, 241) = 19.35, < .001
White-passing (*n*, %)^	47 94.0%	38 80.9%	40 80.0%	42 93.3%	38 77.6%	205 85.1%	χ^2^ (4, 241) = 10.48, *p* = 0.033

Analysis of Variance (ANOVA) was run on log(10) transformed Fan and Play data, as these were highly positively skewed. Superscripts indicate which groups are significantly different from one another per Tukey post-hoc tests. Chi-square analyses were run on the demographic data followed by cell-comparisons based on standardized adjusted residuals

*ANA* #anarecovery. *ARFID* #arfidrecovery, *BED* #bedrecovery, *MIA* #miarecovery, *ORTHO* #orthorexiarecovery

*Statistically significant group differences at *p* < .05 level after application of the Benjamini–Hochberg procedure

^marginally significant group differences at *p* < .1. After the Benjamini–Hochberg procedure, the highest p-value considered statistically significant was *p* = 0.016, and the highest p-value considered marginally significant was *p* = 0.078. For cell comparisons using Z-scores, a significant *p*-value was set to *p* < .05. We only assessed cell comparisons in cases when the omnibus test was significant after the Benjamini–Hochberg procedure. Fishers Exact Tests were used if any expected cell count was less than 5

**Table 2:** Diagnostic hashtag theme and code totals and code group differences

ED behaviors	ANA	ARFID	BED	MIA	ORTHO	Total	Significance
Dietary restriction (*n*, %)	8, 16.0%	10, 21.3%	11, 22.0%	8, 17.8%	16, 32.7%	53	χ^2^ (4, 241) = 4.75, *p* = 0.314
Binge eating (*n*, %)*	0, 0%*	0, 0%*	27, 54.0%*	4, 8.9%	1, 2.0%*	32	χ^2^ (4, 241) = 92.98, *p* < .001
Compensatory behaviors (*n*, %)*	2, 4.0%	0, 0%*	3, 6.0%	9, 20.0%*	3, 6.1%	17	χ^2^ (4, 241) = 15.93, *p* = 0.003
*Theme occurrence*							
**Centrality of food to ED and recovery**	31	39	38	27	32	167	
Eating/food discussed (*n*, %)*	31, 62.0%	38, 80.9%	38, 76.0%	23, 51.1%	30, 61.2%	160	χ^2^ (4, 241) = 12.20, *p* = 0.016
Food visible (*n*, %) ^	19, 38.0%	23, 48.9%	22, 44.0%	11, 24.4%	14, 28.6%	89	χ^2^ (4, 241) = 8.49, *p* = 0.075
Eating visible (*n*, %)	11, 22.4%	16, 34.0%	10, 20.0%	7, 15.6%	7, 14.3%	51	χ^2^ (4, 241) = 6.98, *p* = 0.137
FDOE (*n*, %)*	9, 18.0%^	3, 6.4%	10, 20.0%*	1, 2.2%	1, 2.0%^	24	Fishers = 15.57, *p* = 0.002
Fear Food (*n*, %)*	9, 18.0%	22, 46.8%*	3, 6.0%^	7, 15.6%	9, 18.4%	50	χ^2^ (4, 241) = 27.16, *p* < .001
**What eating disorders look and feel like**	26	23	20	29	36	134	
Explaining EDs (*n*, %)^	16, 32.0%	15, 31.9%	14, 28.0%	18, 20.2%	26, 53.1%	89	χ^2^ (4, 241) = 8.40, *p* = 0.078
Gallows humor (*n*, %)*	22, 44.0%*	10, 21.3%	7, 14.0%*	13, 28.9%	18, 36.7%	70	χ^2^ (4, 241) = 13.70, *p* = 0.008
Personification of EDs (*n*, %)*	1, 2.0%	3, 6.4%	1, 2.0%	12, 26.7%*	4, 8.2%	21	Fishers = 18.97, *p* < .001
**Recovery as process**	40	39	38	40	37	194	
Me then/me now (*n*, %)*	13, 26.0%	6, 12.8%*	18, 36.0%	14, 31.1%	23, 46.9%*	74	χ^2^ (4, 241) = 14.360, *p* = 0.006
Recovery is going well (*n*, %)	21, 42.0%	20, 42.6%	26, 52.0%	18, 40.0%	19, 38.8%	104	χ^2^ (4, 241) = 2.19, *p* = 0.706
Recovery is a struggle (*n*, %)	21, 42.0%	24, 51.1%	16, 32.0%	23, 51.1%	15, 30.6%	99	χ^2^ (4, 241) = 7.75, *p* = 0.101
**Getting and giving help**	22	27	28	26	19	122	
Recovery tips (*n*, %)*	4, 8.0%	5, 10.6%	17, 34.0%*	7, 15.6%	6, 12.2%	39	χ^2^ (4, 241) = 15.81, *p* = 0.003
Showing support (*n*, %)	8, 16.0%	11, 23.4%	8, 16.0%	10, 22.2%	9, 18.4%	46	χ^2^ (4, 241) = 1.49, *p* = 0.830
Trigger warning (*n*, %)^	2, 4.0%	8, 17.0%	5, 10.0%	10, 22.2%	4, 8.2%	29	χ^2^ (4, 241) = 9.46, *p* = 0.049
Treatment (*n*, %)^	15, 30.0%	13, 27.7%	6, 12.2%	6, 13.3%	7, 14.3%	47	χ^2^ (4, 241) = 9.06, *p* = 0.059
Inpatient storytime (*n*, %)^	8, 16.0%	3, 6.5%	1, 2.0%	4, 8.9%	1, 2.0%	17	Fishers = 8.94, *p* = 0.045
**Negotiating diet culture**	17	9	32	14	35	107	
Diet culture critique (*n*, %)*	8, 16.0%	6, 12.8%^	11, 22.0%	6, 13.3%^	31, 63.3%*	62	χ^2^ (4, 241) = 46.72, *p* < .001
Diet culture promotion (*n*, %)*	3, 6.0%	1, 2.1%^	20, 40.0*	3, 6.7%	1, 2.0%*	28	χ^2^ (4, 241) = 50.34, *p* < .001

*ANA* #anarecovery, *ARFID* #arfidrecovery, *BED* #bedrecovery, *MIA* #miarecovery, *ORTHO* #orthorexiarecovery, *ED* eating disorder, *FDOE* full day of eating

*Statistically significant group differences at *p* < .05 level after application of the Benjamini–Hochberg procedure

^Marginally significant group differences at *p* < .1. After the Benjamini–Hochberg procedure, the highest p-value considered statistically significant was *p* = 0.016, and the highest *p*-value considered marginally significant was *p* = 0.078. For cell comparisons using Z-scores, a significant *p*-value was set to *p* < 0.05. We only assessed cell comparisons in cases when the omnibus test was significant after the Benjamini–Hochberg procedure. Fishers Exact Tests were used if any expected cell count was less than 5

In addition to engagement data, the coding team noted whether creators appeared to be female-presenting or white-passing when it was apparent.^1^ 213 of the videos (88.4%) had a female-presenting content creator; 204 (84.6%) of the videos had white-passing content creators. In 17 (7.1%) of the videos, the content creator was not visible in the video. There was a statistically significant difference in the number of female-presenting creators across diagnostic hashtags [χ^2^ (4, 241) = 19.35, *p* < 0.001], such that the number of female-presenting creators in the #arfidrecovery group was significantly lower than would be expected by chance (Z = −4.3, *p* < 0.001). There was a marginally statistically significant difference in the number of white-passing creators across diagnostic hashtags [χ^2^ (4, 241) = 10.48, *p* = 0.033], with trends toward there being less white-passing creators in the #orthorexiarecovery group and more in the #anarecovery group than would be expected by chance.

Across the videos, we tracked mentions of the following eating disorder behaviors: dietary restriction, binge eating, and compensatory behaviors. The most frequently mentioned symptom across all diagnostic hashtags was dietary restriction, which showed up in nearly a quarter (22.0%) of the videos. There was no statistically significant difference in the number of dietary restriction mentions across diagnostic hashtags [χ^2^ (4, 241) = 4.75, *p* = 0.314]. There was a statistically significant difference in the number of binge eating mentions across diagnostic hashtags [χ^2^ (4, 241) = 92.98, *p* < 0.001], such that #bedrecovery had more (Z = 9.5, *p* < 0.001), while #anarecovery (Z = − 3.1, *p* = 0.002), #orthorexiarecovery (Z = − 2.6, *p* = 0.009) and #arfidrecovery (Z = − 3.0, *p* = 0.003) had fewer mentions than would be expected by chance. There was also a statistically significant difference in the number of compensatory behavior mentions across diagnostic hashtags (χ^2^ (4, 241) = 15.93, *p* = 0.003), such that #miarecovery had more mentions (Z = 3.8, *p* < 0.001), while #arfidrecovery had fewer mentions (Z = − 2.1, *p* = 0.036) than would be expected by chance. See Table 2 for further information on diagnostic hashtag group comparisons.

### Themes

This section details the following five major themes we identified in the videos: *centrality of food to eating disorder and recovery*, *what eating disorders look and feel like*, *recovery as a process*, *getting and giving help*, and *negotiating diet culture in recovery*. Each video had a mean of 3.00 of these major themes (*sd* = 1.00). According to Chi-square tests, there were no significant associations between any of the themes. In the following section, for each theme, we first describe its overarching meaning and then describe codes that were salient in generating that theme. Throughout, we offer statistical comparisons of the code prevalence between the diagnostic hashtags. See Additional file 1: Appendix A for a list of all codes along with their definitions. See Table 2 for frequencies of theme and code occurrence across the sample and between diagnostic hashtag groups.

#### Centrality of food to eating disorders and recovery

Showing or discussing food or eating habits was a primary way in which creators communicated both their eating disorder and recovery experiences. 167 (69.30%) posts included content centering food or eating, as demonstrated by the codes described below.

*Food and Eating Discussed or Displayed*. Food and eating were explicitly mentioned in 160 (66.39%) of the videos. Some of these incorporated visible food (*n* = 89, 36.93%) or visible eating (*n* = 51, 21.16%). There were significant differences in the number of videos that *discussed food or eating* across the diagnostic hashtags [χ^2^ (4, 241) = 12.20, *p* = 0.016] with #arfidrecovery videos including more (Z = 2.3, *p* = 0.021) and #miarecovery videos including less of this code than would be expected by chance (Z = − 2.4, *p* = 0.016). There were only marginally significant differences in the presence of food being shown on camera [χ^2^ (4, 241) = 8.49, *p* = 0.075], with trends toward #miarecovery showing it less and #arfidrecovery more than would be expected by chance. By contrast there were no significant differences in the presence of eating on camera across the different hashtags [χ^2^ (4, 241) = 6.98, *p* = 0.137].

*Full Day of Eating*. A number of the videos that visually included food did so as part of the “what i eat in a day” (WIAIED) or *“full day of eating” (FDOE)* format in which content creators documented all of the meals and snacks they consumed over the course of one day. FDOE videos were 9.96% of the total sample (*n* = 24). In some *FDOE* videos, creators showed imagery of this food, while in others they filmed themselves actually eating each of their meals throughout the day. Creators sometimes showed themselves cooking meals with commentary like, “I decided to cook my first meal of the day.” Other examples included creators saying, “This is what I ate today in ‘all in’ bulimia recovery; day 50.” There were significant differences in the inclusion of *FDOE* videos across the diagnostic hashtags [χ^2^ (4, N = 241) = 16.328, *p* = 0.003]. #anarecovery and #bedrecovery included this code significantly more than would be expected (Z = 2.1, *p* = 0.036 and Z = 2.7, *p* = 0.007 respectively) by chance, while #orthorexiarecovery included it less (Z = − 2.1, *p* = 0.036).

**Fig. 3:**
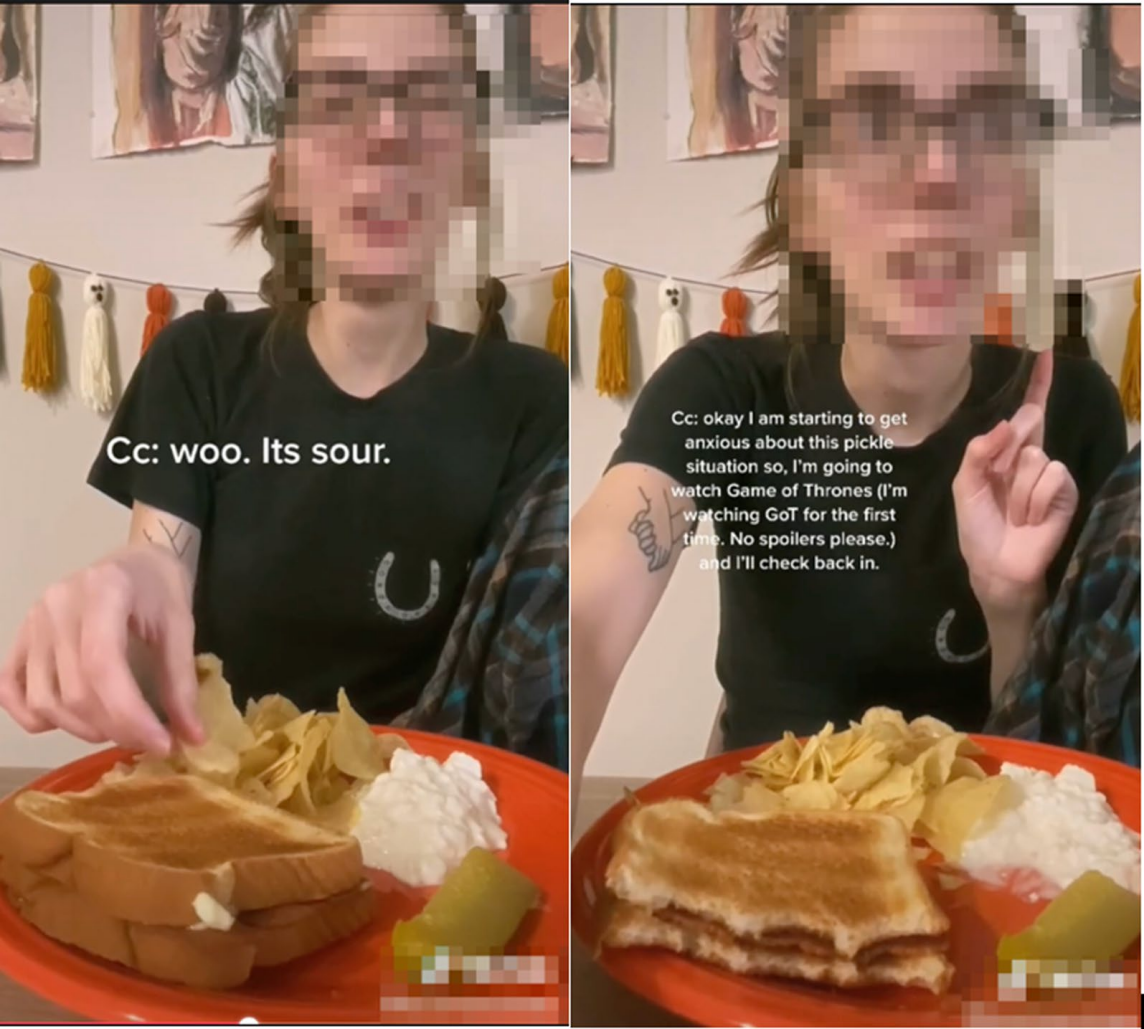
Screenshots of video in which the content creator shares her experience with a fear food

*Fear Foods*. Fear food videos involved individuals discussing food that currently or previously posed a challenge for them to eat without engaging in compensatory behaviors or triggering a binge eating response. *Fear foods* were mentioned among 20.75% of the videos (*n* = 50). Some of these showed individuals challenging themselves to eat a fear food on camera, often depicting significant struggle. For example, one creator filmed herself eating a pickle (what she identified as a fear food) and overlaid text narrating her experience and strategies for successfully conquering her fear (See Fig. 3). Others shared pictures of their fear foods to show their progress in recovery. There were significant differences in the presence of *fear food* content across diagnostic hashtags [χ^2^ (4, 241) = 27.16, *p* < 0.001]. #arfidrecovery videos included this code more than would be expected by chance (Z = 4.9, *p* < 0.001) and #bedrecovery videos included it less than would be expected (Z = − 2.9, *p* = 0.004).

#### What eating disorders look and feel like

There were a substantial number of videos that focused on explaining what eating disorders look and feel like. Some of this material appeared to be directed at outsiders who might not know what eating disorders are or could have misconceptions that content creators aimed to correct. For other types of content, the audience was less clear but appeared to target individuals with some level of insider knowledge. In these videos, content creators often focused on enacting experiences they had while in the grips of their eating disorder. This theme was present among 134 (55.60%) of the videos.

**Fig. 4:**
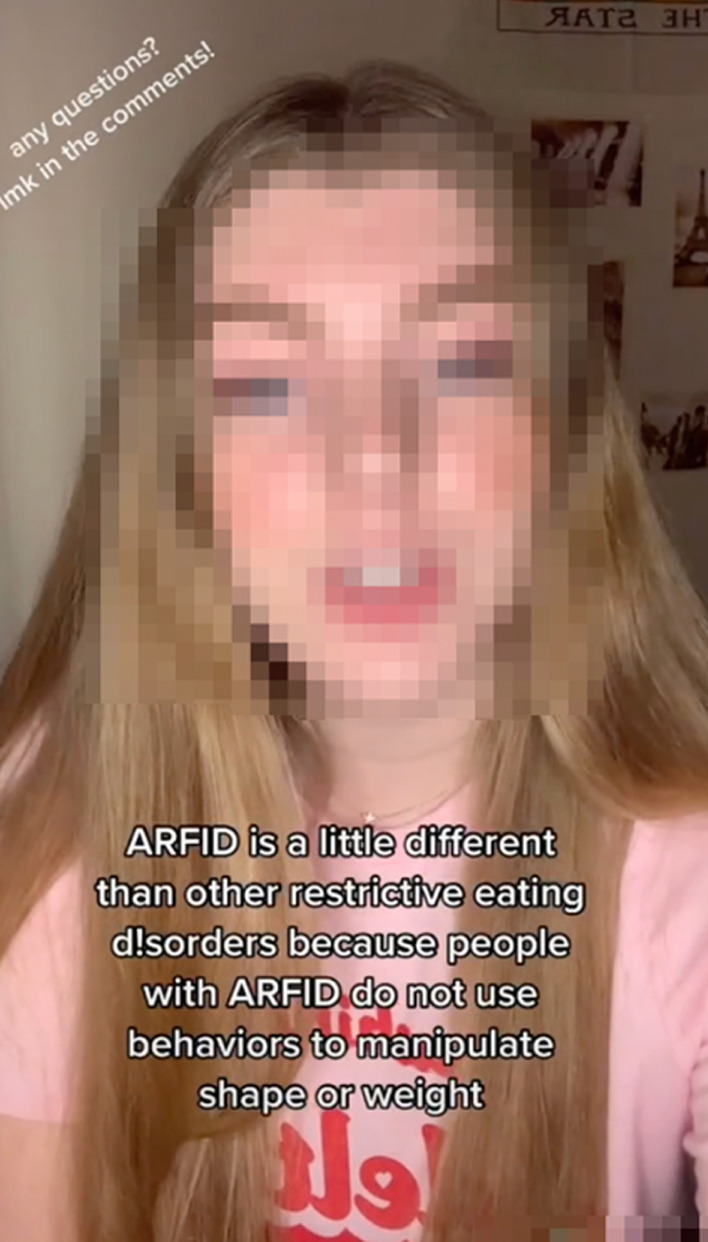
Screenshot from video in which the content creator defines ARFID for the audience

*Explaining Eating Disorders*. 89 (36.93%) videos focused on correcting misconceptions about eating disorders or trying to raise awareness about what these conditions look like and how severe they can be. Some also informed viewers of the distinctions between the various diagnoses, often including text and spoken definitions of eating disorders. For example, in one video, the content creator defined ARFID with an explanatory text superimposed over a video that showed her challenging a fear food: “ARFID is Avoidant Restrictive Food Intake Disorder. It is a non-body-image based eating disorder” (See Fig. 4). Other videos were less flatly informational and instead emphasized the danger and suffering of having an eating disorder as a way of stressing their severity and combatting their glamorization. For example, one video communicated this harm by “sharing all the dangerous things I did when I was struggling with bulimia.”

Some content creators pushed back against assumptions that only those with low-weight body sizes suffered from eating disorders. Others critiqued social food norms that encourage dieting or promote thin ideals. One creator’s video accomplished this by discussing their socialized food norms and visually displaying a compilation of “foods I used to convince myself I liked when I had an eating disorder:” such foods included cauliflower rice and other low-calorie foods. There were marginally significant differences in the presence of the *explaining eating disorders* code across diagnostic hashtags [χ^2^ (4, 241) = 8.40, *p* = 0.078], with trends toward #orthorexiarecovery posts including in more than would be expected by chance.

*Gallows Humor*. Gallows humor, defined by Herrick and colleagues [39] as “making fun of a life-threatening, disastrous, or terrifying situation,” was another common way in which content creators communicated what having an eating disorder feels like. An example of the use of *gallows humor* was exemplified by one creator posting a video of herself with an upbeat, humorous song playing in the background with in-video text that read: “When u go out to town and ur 6 drinks in and suddenly telling everyone ur an0rexic.” The overall air of the video was humorous and light, but the content being shared communicated something more intrinsically damaging to the experience of recovery. Another example of *gallows humor* was a video in which the content creator showed herself in bed with a feeding tube holding a large, wrapped chocolate rabbit. The on-screen text annotated this scene by pointing to the irony of her mom gifting her this “treat” while in an inpatient setting, thus completely misunderstanding the severity and nature of her eating disorder. There were significant differences in the use of *gallows humor* across diagnostic hashtags [χ^2^ (4, 241) = 13.70, *p* = 0.008]. #anarecovery videos were significantly more likely (Z = 2.6, *p* = 0.009) while #bedrecovery (Z = -2.6, *p* = 0.009) videos were significantly less likely to use gallows humor than would be expected by chance.

**Fig. 5:**
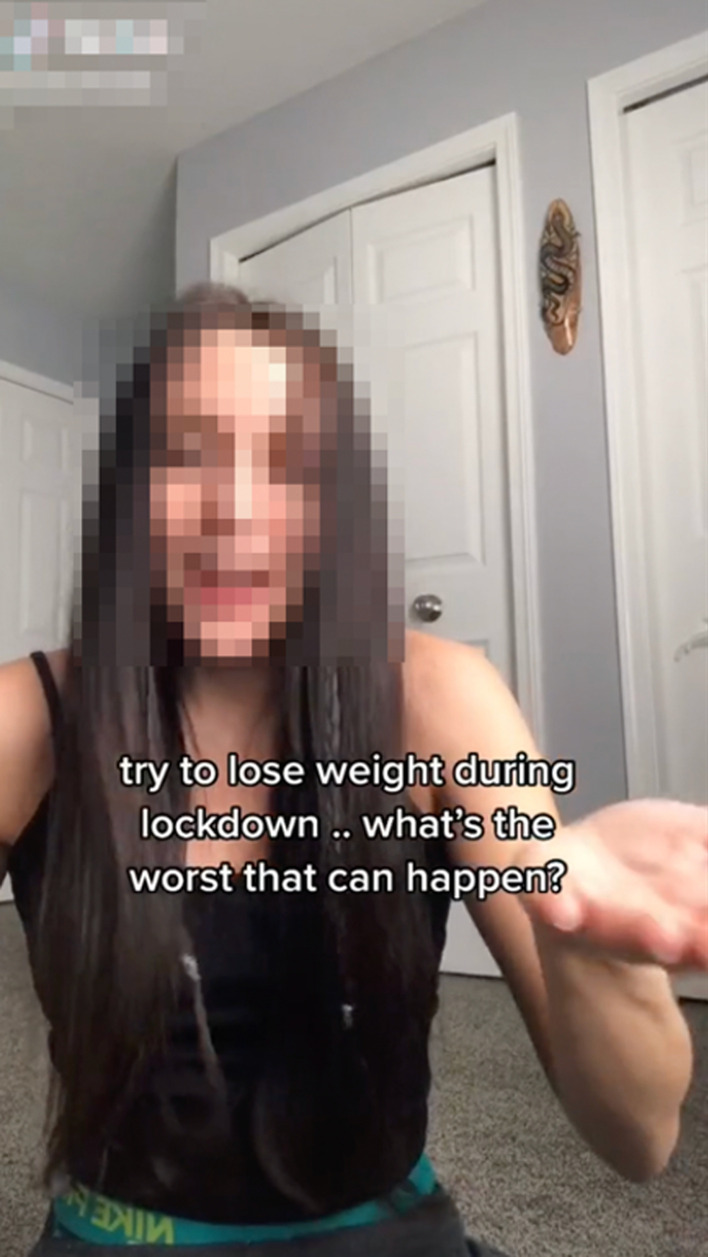
Screenshot from a video in which the content creator comically dramatizes shaking hands with her eating disorder

*Personification*. *Personification* was another way in which content creators strove to capture on camera what eating disorders look and feel like. These posts portrayed or described eating disorders as friends, companions, or enemies. Some of these coincided with *gallows humor.* For example, one creator dramatized shaking hands with her eating disorder in the video while stating: “try to lose weight during lockdown…what’s the worst that can happen?” (See Fig. 5). Other examples were much more sobering: one creator documented compensatory acts that their “eating disorder made them do,” thereby externalizing eating disorder behaviors and urges as forced on them by an outward identity. There were significant differences in the use of *personification* across the diagnostic hashtags (Fishers = 18.97, *p* < 0.001). Videos tagged with #miarecovery included this code more than would be expected (Z = 4.7, *p* < 0.001) by chance.

#### Recovery as a process

Many (*n* = 194; 80.50%) videos used storytelling techniques to relay their creator’s recovery processes progressing over time with a range of highs and lows. These narratives utilized video cuts, still photographs, rhetorical choices in captions and overlaid text, and audio with the creator’s own voice to convey that the creator’s experience of recovery was in flux and had changed over time.

**Fig. 6:**
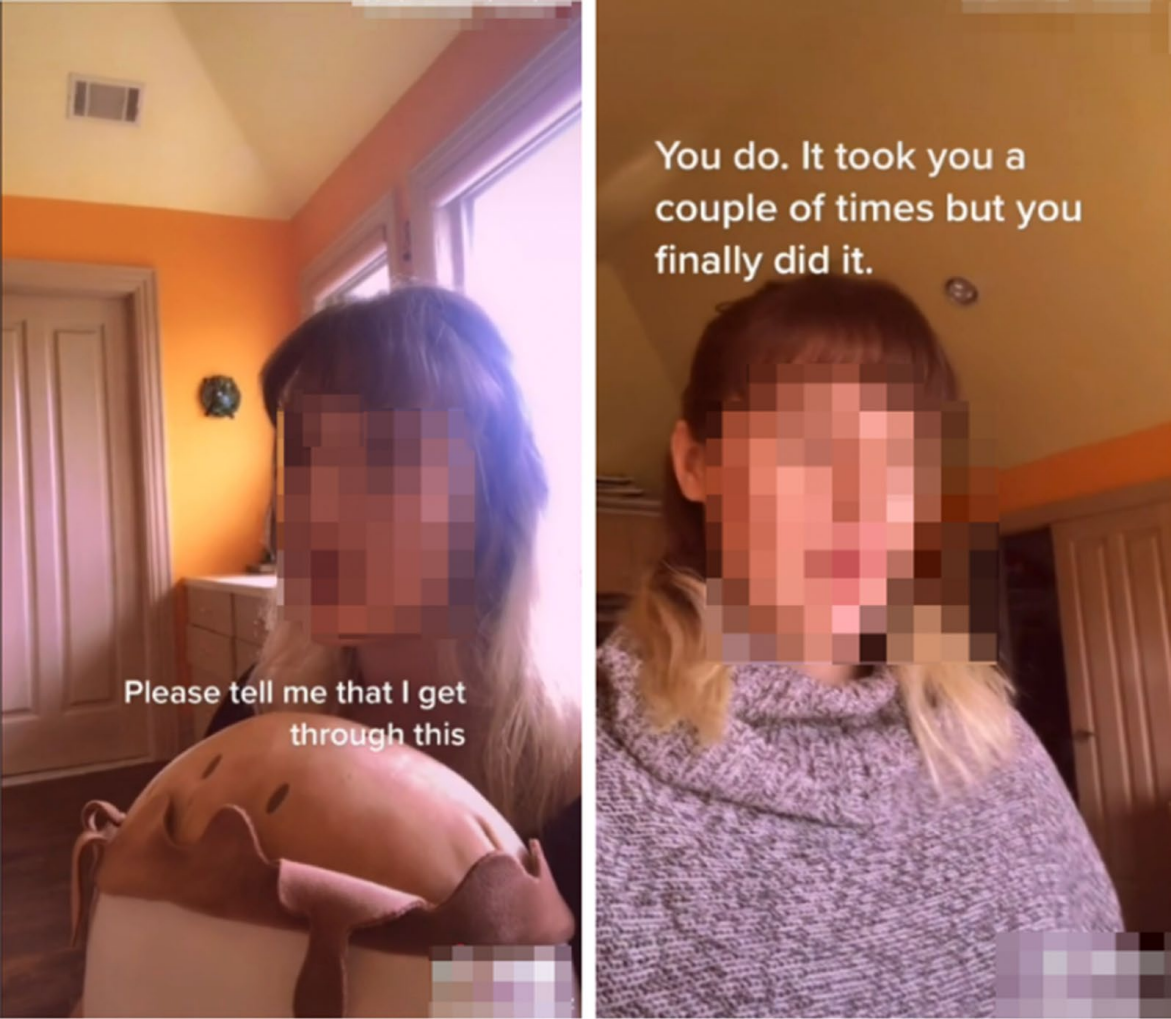
Screenshots from a video in which the content creator stages a dialogue between her past and present self

*Me Then and Me Now*. 74 (30.71%) videos featured creators performing as “me then” and “me now,” offering depictions both of their present self and a version of their past self. To do this, videos often used cuts to place the two selves in dialogue with each other. For example, one creator performed her past self desperately demanding of her present self, “Please tell me I get through this.” The video then cut to her present self who affirmatively responded, “You do. It took you a couple of times but you finally did it” (See Fig. 6). This juxtaposition of past and present self within several seconds conveyed hope and reinforced the message in the caption, “Recovery from Bulimia really is possible.” Other videos achieved similar effects by overlaying photographs of the creator in earlier moments of their recovery journey with text or audio dialogue of encouragement: “Sometimes it gets better.”

Some videos presented changes from their past self through humor, accentuated by TikTok cuts and audio memes, as in one where the creator was idly typing with a text overlay saying, “Remember when you were hardcore vegan and shamed everyone who wasn’t?” switching to numerous cuts of the same person wincing dramatically to the beat of a popular, upbeat song. This dramatized interaction captured their journey as a process of leaving a shameful or embarrassing past eating disordered self behind. There were significant differences in the use of the *me then/me now* formula across diagnostic hashtags [χ^2^ (4, 241) = 14.360, *p* = 0.006]. Videos tagged with #orthorexianervosa were more likely (Z = 2.8, *p* = 0.005) and #arfidrecovery videos were less likely (Z = − 3.0, *p* = 0.003) to use it than would be expected by chance.

*Recovery is Going Well*. 104 (43.15%) videos portrayed to the audience that recovery was “going well.” These videos would share moments that depicted the creator conquering something challenging within their eating disorder. Additionally, “going well” videos sometimes portrayed smaller positive movements towards recovery, like starting over the recovery process after a relapse. One creator shared a video of herself eating what might be considered “unhealthy food” in mainstream diet culture with in-video text reading “So today I had a huge recovery win … food is fuel!” This communicated to viewers that, from her perspective, her recovery was “going well” and something to celebrate. There were no significant differences in the presence of the *recovery is going well* code across diagnostic hashtags [χ^2^ (4, 241) = 2.19, *p* = 0.706].

*Recovery is a Struggle*. 99 (41.08%) videos signaled that recovery “is a struggle” by sharing, for example, relapses, advice they wish they had known during their eating disorder and eating disorder related behaviors the creator could not get over. One creator cried on camera while using overlaid text to reflect on how long she had been dealing with her eating disorder: “I wish I could go back to a time before my ED. When I wasn’t so hyper-aware and harsh towards my body. When I didn’t care about what I ate or the amount of calories I was consuming. I wonder what my younger self would think of me now. I wish it would all just go away. It gets so tiring.” In this case, the creator shared their journey towards recovery by doing away with the narrative arc of feeling “cured” and instead foregrounding the ongoing struggle of recovering. There were no significant differences in the presence of the *recovery is a struggle* code across diagnostic hashtags [χ^2^ (4, 241) = 7.75, *p* = 0.101].

#### Getting and giving help

122 (50.62%) of the posts across the dataset focused on getting help for one’s eating disorder or helping others who were still struggling or in the process of recovery. Helping others often took the form of explicitly showing support or affirming the journey of other community members, sharing tips to help others in their recovery, or making videos with “trigger warnings” in order to keep the community a safe space for individuals who might be vulnerable to particular kinds of eating disorder related content. Additionally, content creators discussed the ways in which they had reached out for professional help or were currently undergoing treatment both in an inpatient setting and through other healthcare providers such as therapists or nutritionists.

*Recovery Tips*. 39 (16.18%) videos offered concrete help and coping methods geared toward confronting fear foods, following a meal plan, and avoiding binging. One creator, for example, offered tips relevant to her imagined audience of individuals also going through recovery: “8 foods I ate to recover from my eating disorder.” There were significant differences in the presence of *recovery tips* across diagnostic hashtags [χ^2^ (4, 241) = 15.81, *p* = 0.003]. Videos tagged with #bedrecovery included this code more than would be expected (Z = 3.8, *p* < 0.001) by chance. Many of the tips included among BED videos encouraged weight loss techniques, such as volume eating and examples of high protein/low-calorie foods.

*Showing Support*. 46 videos (19.09%) placed themselves in direct dialogue with other pro-recovery creators and their audiences to offer support and advice. This included interweaving their videos with those belonging to other creators (“stitching”), placing comments from past posts in their video, or juxtaposing videos with another creator’s videos (“dueting”). One creator “stitched” a comment a user had made on one of her previous posts: “this might sound like a stupid question, but how can I tell the difference between binging and eating snacks?” before then going on to answer the question in the video. This online technique of “stitching” a comment directly engages a viewer who might not have received support or tips for recovery before. Across diagnoses, creators showed support to other content creators by responding to their videos with stitches. Frequently, videos opened with part of another individuals’ post where they expressed an opinion or a struggle, and then cut to the creator who then offered encouragement, affirmations, and replies to that content. Duets included, for example, creators dancing side-by-side in their respective videos as a mode of celebrating recovery in community. There were no significant differences in the presence of the *showing support* code across diagnostic hashtags [χ^2^ (4, 241) = 1.49, *p* = 0.830].

*Trigger warnings*. *Trigger warnings* appeared infrequently (*n* = 29, 12.03%) as an additional cue indicating content creators’ awareness of an audience that was potentially recovering from or still struggling with an eating disorder. One creator shared a trigger warning in their video discreetly as: “Eating Dis0rder #triggerwarning.” Another way videos included trigger warnings was by featuring it as large, bold text at the beginning of the video. There were marginally significant differences in the presence of *trigger warnings* across diagnostic hashtags [χ^2^ (4, 241) = 9.46, *p* = 0.049] with trends toward #miarecovery and #arfidrecovery having more trigger warnings than would be expected by chance.

*Treatment*. Treatment was sometimes mentioned in videos (*n* = 47, 19.50%) as part of the recovery journey. Discussion normally centered achieving or struggling with treatment, particularly the struggles or achievements of working with treatment goals and providers. These videos often included successfully eating or continuing to struggle with fear foods and nutrition goals. Some discussed the absence of adequate treatment or relayed difficult discussions with providers who were not meeting their needs. There were marginally significant differences in the presence of the *treatment* code across diagnostic hashtags [χ^2^ (4, 241) = 9.06, *p* = 0.059] with trends toward #anarecovery including it more than would be expected by chance.

**Fig. 7:**
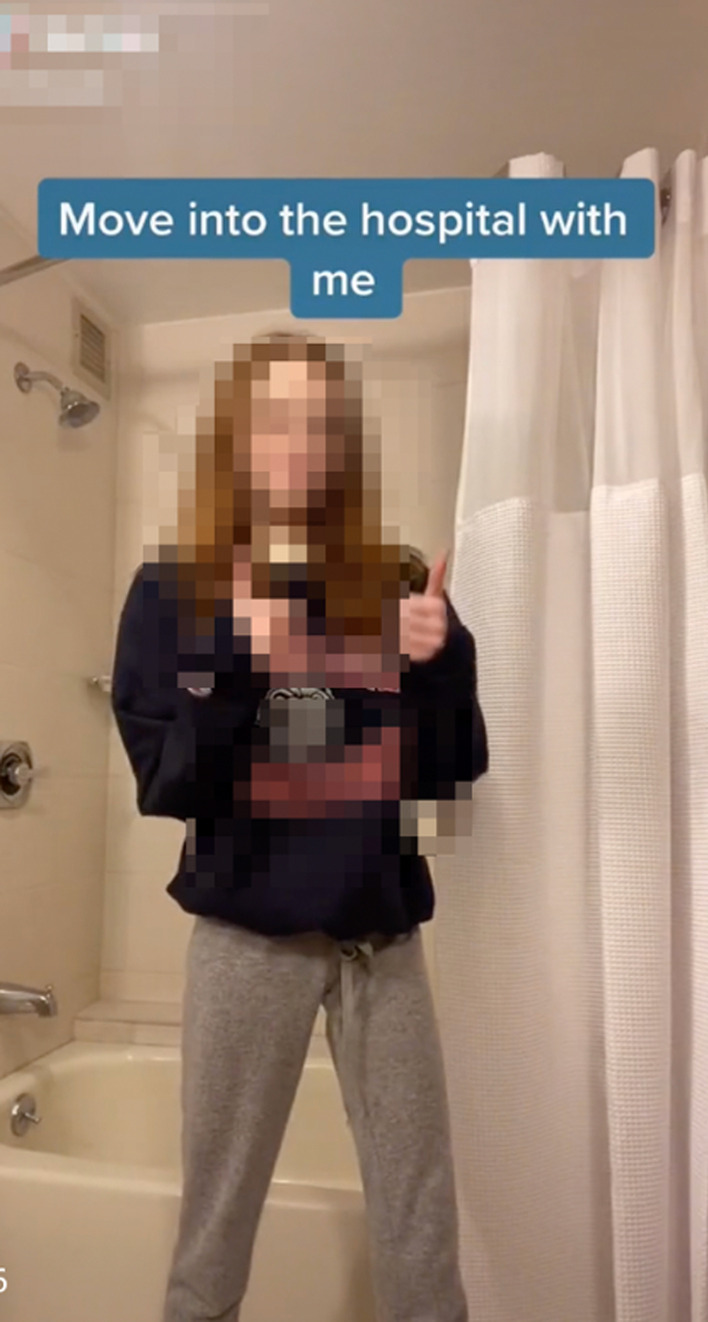
Screenshot from a video in which content creator films her first day in inpatient treatment

*Inpatient Storytime*. 17 videos (7.05%) took the form of *inpatient storytime* [39]. In *inpatient storytime* videos, individuals displayed their daily experience in eating disorder inpatient facilities, often including detailed photographs or video cuts of their activities, treatment groups, and mealtimes. Some of these videos seemed to take an audience on a tour of the inpatient experience by portraying a “day in the life” or framing the post with titles like “move into the hospital with me” (Fig. 7) Sometimes these videos more subtly featured feeding tubes, IV’s, and hospital rooms in the background while creators discussed other experiences. Other portrayals of inpatient treatment were more graphic. For example, in one video the content creator pulled out her feeding tube on camera to signal her progress in recovery. There were marginally significant differences in the use of the *inpatient storytime* across the groups (Fishers Exact = 8.94, *p* = 0.045) with trends toward #anarecovery videos including it more than would be expected by chance.

#### Diet culture in recovery

Diet culture was notably present in the pro-recovery TikTok space (*n* = 107, 44.40%). Many videos focused on critiquing diet culture and linking problematic standards around beauty and thinness to the development of eating disorders. A number even explicitly called out ideas such as fatphobia or weight stigma. On the other hand, other creators promoted diet culture by emphasizing weight loss as a key to recovery, documenting restrictive eating, valorizing the thin body, and/or shaming larger bodies.

*Diet Culture Critique*. Diet culture critique videos (*n* = 62, 25.73%) included individuals speaking about their experiences rejecting dieting or undermining the labeling of foods as good or bad. In one example of diet culture critique, a creator mocked a common phrase that values thinness over fueling your body by re-phrasing it: “Our new catch phrase … Nothing feels as good as milkshakes taste” (a play on model Kate Moss’s quote “nothing tastes as good as skinny feels” that is a staple in pro-ED communities). Another creator normalized their food choices by refusing to assign the label of good or bad to what she eats: “every day is different and all foods fit in my day.” Some of these videos also dealt with weight stigma, stressing that “skinny does not equal healthy” or using the word “fat” as a celebratory identity. For example, one creator sang along to the lyric “I got a perfect body” in the Regina Spektor song “Folding Chair.” Some creators more directly tackled how the problem of weight stigma in medicine and society more generally caused friction with their attempts to recover from their eating disorder. For example, one content creator critically recounted receiving diet advice from doctors while seeking help with their eating disorder.

There were significant differences in the presence of *diet culture critique* across diagnostic hashtags [χ^2^ (4, 241) = 46.72, *p* < 0.001].Videos tagged with #orthorexiarecovery were significantly more likely to include diet culture critique than would be expected by chance (Z = 6.7, p < 0.001); whereas videos tagged with #miarecovery or #arfidecovery were less likely (Z = − 2.1, *p* = 0.036 and Z = − 2.3, *p* = 0.021 respectively).

**Fig. 8:**
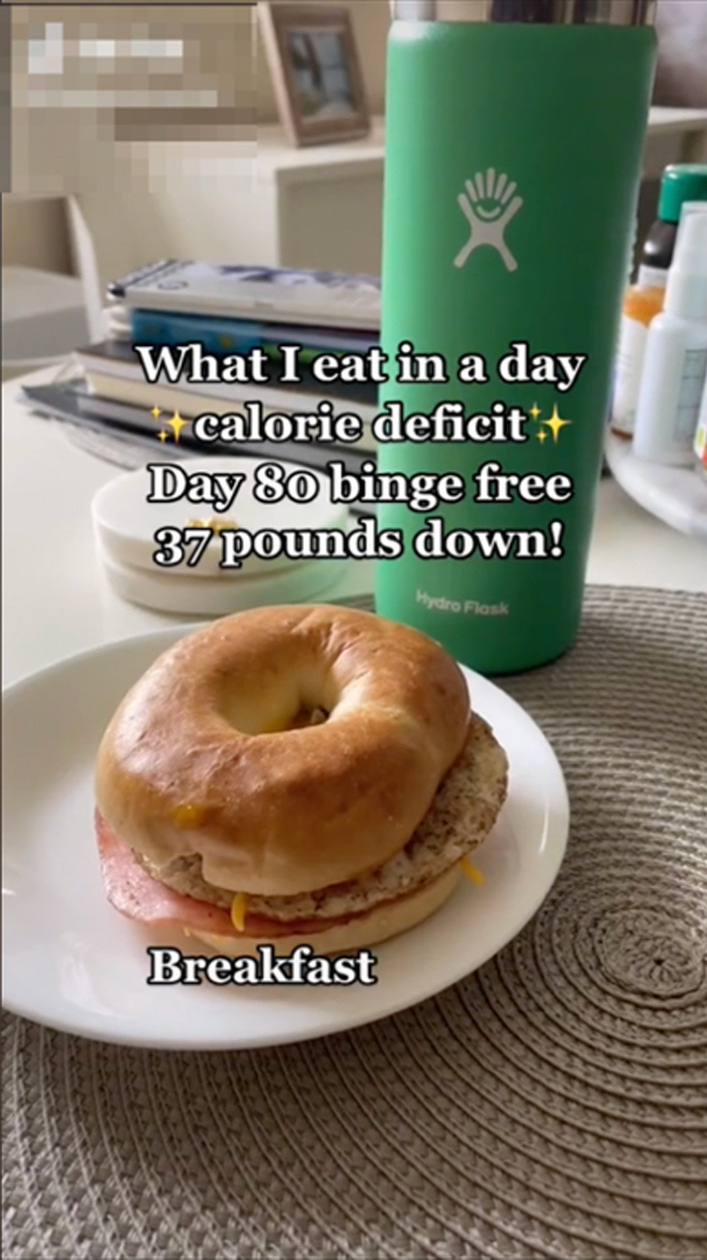
Screenshot from a FDOE video that contains diet culture promotion such as discussions of weight loss and caloric restriction

*Diet Culture Promotion*. In contrast, diet culture promotion videos (*n* = 28, 11.62%) consisted of individuals speaking about losing weight and restrictive diets (Fig. 8). For example, a creator described losing a significant amount of weight in their caption: “265 pounds down #bariatricsurgery,” while another creator showcased a prescribed restrictive diet for her binge eating disorder: “My doctor just recommended that I start a 1200 cal diet which means that there is going to be a lot of changes …These are what I use…” Diet culture promotion also consisted of creators quantifying calories and portions of what they were eating. One creator described the caloric value in some snack boxes that she created to avoid overeating: “They've got different things in them… this one is 97 cal, these are about 70, these 99.”

Negative body display also indicated the promotion of diet culture. For example, one creator presented their body with minimal clothing in front of the camera with self-deprecating words drawn on their skin and grabbing their stomach. This was followed by a compilation of the creator eating various foods that would normally be deemed unhealthy (e.g. pizza and fried chicken). The creator’s caption reads: “I gained 70 lbs over 4 years due to binge eating. It’s been a long road, but proud to say that’s in my past now.” This creator further makes the connection that their choices in types of food and the amount of weight they gained was the main source of their disorder and that weight loss was the focus of recovery. There were significant differences in the presence of diet culture promotion across diagnostic hashtags [χ^2^ (4, 241) = 50.34, *p* < 0.001]. Videos tagged with #bedrecovery were significantly more likely to include diet culture promotion than would be expected by chance (Z = 7, *p* < 0.001). Videos tagged with #orthorexiarecovery and #arfidrecovery were less likely to include *diet culture promotion* than would be expected (Z = − 2.3, *p* = 0.021 for both).

## Discussion

Across the entire sample of videos there are a number of consequential findings. The homogeneity of the kinds of individuals represented, tending towards female-presenting and white-passing content creators, suggest that the diversity of individuals struggling with eating disorders may not see themselves in these pro-recovery communities. This resonates with scholarship pointing to the widespread SWAG (skinny, white, affluent, girl) stereotype of eating disorders that tends to erase other identities in popular imaginations and even clinical settings [61]. It also reproduces Au and Cosh’s [36] identification of “underrepresentation” as one potentially problematic aspect of pro-recovery Instagram. Alternatively, individuals with marginalized identities may be coalescing around different sets of hashtags and creating communities elsewhere. Further research is needed to better understand the full ecosystem of eating disorder support communities and hashtags online.

To the authors’ knowledge, this is the first study to compare pro-recovery communities online across different diagnostic self-identifications. The fact that there are substantial variations between these hashtags suggests that this is a needed area of research and that pro-recovery communities are not homogenous in their presentation of recovery. Diagnostic labels appear to influence how online content creators present their experiences of their eating disorder through their videos and may be integrated into an individual’s perception of their illness. The videos analyzed seemed to respond to social imaginings of different eating disorder diagnoses, presenting a range of visions of what eating disorders and eating disorder recovery look like to the large audience viewing them. Such imaginings may play a critical role in differentially shaping individuals’ understanding of how to pursue recovery or what treatment for their eating disorder will look like based on their own diagnostic self-identification.

### Diagnosis and recovery portrayal

One key difference between the hashtags was in the rhetorical tools content creators used to describe what their eating disorders look and feel like. Variations in the use of *gallows humor* between the hashtags was especially striking. While *gallows humor* was among the most prevalent codes in the dataset, #anarecovery and #orthorexiarecovery videos featured more gallows humor, while #bedrecovery featured almost none. This disparity may indicate the presence of a social norm around who is allowed to present their experience with humor or who has an illness that is legible enough such that others can understand the “darkness” or gallows element of their jokes.

The most referenced symptom across diagnostic hashtags was dietary restriction. This aligns with literature on the central role of dietary restriction across all eating disorders [62]. Relatedly, eating and food discussions of different sorts (*food/eating discussion or display, FDOE,* and *fear foods*) was present across posts at fairly high frequencies. As such, many creators seemed to communicate an understanding that eating and food is a central part of eating disorder recovery. That said, there were differences in how food was presented or described; #bedrecovery posts were particularly likely to include *FDOE* posts, centering an aspect of accountability around eating. As shame and embarrassment in eating around others as well as eating large quantities of food in secret are clinical features of BED [6], this public display of eating may have been a way of explicitly countering these disordered eating tendencies. Additionally, #arfidrecovery posts were more prone to discussions of *fear foods*. This is likely due to the clinical presentation of ARFID and the hallmark criteria of food avoid­ance based on the sensory characteristics of said food [6].

Notably, despite binge eating being a behavior that is common across eating disorder diagnoses (particularly when including subjective binge eating), binge eating was mentioned infrequently [63]. It surfaced most in the #bedrecovery videos, while it was not mentioned at all in #anarecovery or #arfidrecovery videos and only minimally appeared in the #miarecovery and #orthorexiarecoveryvideos. Considering binge eating is part of BN’s diagnostic criteria,^6^ it is particularly surprising to see the lack of binge eating discussion among the #miarecovery videos. This likely aligns with how treatment centers tend to emphasize disrupting restrictive behaviors and place a smaller focus on disrupting binge eating behaviors outside of BED specific treatment [64]. Furthermore, it may align with an avoidance of discussing binge eating due to the shame associated with it [65].

### Differential treatment

Another pronounced difference across the diagnostic hashtags was the role of treatment in recovery and, along with it, the apparent severity of illness. There were overall fewer mentions of *inpatient storytime* than in Herrick and colleagues’ [39] examination of #EDRecovery. However, the prevalence of this code within the #anarecovery hashtag was comparable to their findings, suggesting that perhaps the videos they examined included a high proportion of individuals recovering from AN. The fact that #orthorexiarecovery had few mentions of *inpatient storytime* or of *treatment* in general has resonances with Valente and colleagues’ [49] findings that identified a tension in ON Twitter communities about whether orthorexia was a social or medical phenomenon.

#bedrecovery posts, like #orthorexiarecovery, rarely mentioned *treatment* of any kind, which may further speak to society’s understanding of BED. While those with BED are more likely to inhabit a larger body [46], popular representations of eating disorders often equate an individual’s level of emaciation with the severity of their illness. As a result, many individuals with BED may not register in the eyes of others (or themselves) as being “sick enough” to receive treatment. Additionally, healthcare providers are more likely to report negative reactions to higher weight patients, and thus could influence providers’ perceptions and their quality of care [66]. This weight bias may lead to increased healthcare avoidance among large-bodied individuals with BED [66], therefore aligning with the lack of inpatient treatment representation seen in our #bedrecovery sample.

### Diet culture in/as recovery

The place of diet culture in these different hashtag communities was another important finding, and one in which there were key differences based on diagnosis. Particularly for those self-identifying with BED and ON, diet culture was a key lens or tool through which they navigated and communicated their recovery experiences. Concretely, #bedrecovery posts included a substantial amount of *diet culture promotion*, which may be linked to pervasive attitudes towards BED as an eating disorder diagnosis. Studies have shown that BED is often seen as a failure to control eating behaviors and a personal choice; whereas, restrictive eating disorders like AN and BN are met with an understanding of the disorder as being further outside of one’s control [43]. Rhetoric about weight loss was particularly common in the #bedrecovery posts. In many ways, recovering from BED was framed as a process of “recovering” from a body that content creators equated as a result of their BED as opposed to being about recovering from actual disordered eating behaviors. Dieting as a way of self-managing and therefore self-treating BED was promoted within this community. Of note, this was not completely homogenous, and 22% of #bedrecovery posts included *diet culture critique*.

In terms of diet culture, #orthorexiarecovery appeared as the inverse of #bedrecovery. 64% of the videos with this hashtag included *diet culture critique***.** In many of these videos, individuals discussed realizing their “healthy eating” had gotten out of control or becoming aware of diet culture as a central element driving their recovery process. In this way, understanding diet culture, coming to terms with its impact, and pushing back against it were often key to how creators documented and enacted their recoveries from ON. Just as diet culture was often presented as the “cure” for BED, rejecting it was depicted as a key to recovery from ON. This could be due to the nature of orthorexia in that its symptomatology often includes hyper-fixation on what society would consider “healthy” foods, a major facet of diet culture [67]. As such, recovery from ON would have to involve a rejection of such notions.

### Intersections of pro-recovery and pro-ED

Finally, this study’s results align with previous work that has questioned the extent to which pro-recovery content actually differs from pro-ED content and the way pro-ED messages infiltrate pro-recovery spaces. Many videos with pro-recovery hashtags contained pro-ED content through diet culture promotion and descriptions of disordered eating behaviors just as caloric restriction. There exists a fine line between the two distinct communities, and this likely puts TikTok users at an increased risk of viewing triggering content if they are seeking support in a pro-recovery online space.

As others have noted [39, 42], representations of recovery often included images of very thin bodies, even if they were presented as a “before” as opposed to an ideal. The visual display of food also contributed to the fine line between pro-ED and pro-recovery. Many of the videos in our dataset contained diet culture and even explicit documentation of the numbers of calories content creators were consuming or being advised to consume, as well as the quantification of their weight loss. As shown by Greene and Brownstone [68], self-quantification is a common feature of pro-ED communities online. Although some videos included trigger warnings, these were overall infrequent. The lack of trigger warnings combined with the TikTok algorithm means it is likely that individuals seeking help in ED recovery on the platform could be exposed to at least some harmful or triggering content.

## Strengths and limitations

Some limitations of the study relate to the sample of posts we analyzed. For example, the findings are rooted in five discrete hashtags that do not necessarily capture the full TikTok pro-recovery community and that draw artificially clear lines between the diagnoses of interest. The sample is also limited because we only examined publicly available videos and excluded videos that were not in English. We also focused primarily on fairly popular videos, so the trends we identified may be different from content by creators with smaller followings. For example, our sampling procedure of taking the most viewed posts per hashtag may have impacted the study results because the themes and symptoms may only represent those more likely to attract a larger viewership. That said, such popular posts may be particular impactful in the Tik Tok space.

Since we chose not to sample posts by content creators identifying as minors to protect potentially vulnerable individuals, our sample does not include content produced by a large subset of the TikTok user base. TikTok’s algorithmic curation of viewer’s “For You” page offers an additional complication to our sample as it may present the more popular videos to viewers outside the lived experience of eating disorders or not seeking pro-recovery hashtags, thereby increasing the viewership of some videos and the prevalence of some themes over others. Finally, since we did not talk with viewers or content creators about their experience, we have limited data on how individuals experience and interpret these posts themselves. On the other hand, strengths of this project include the novelty of cross-diagnostic comparison in discussions of online eating disorder communities and the timeliness of the focus on TikTok. Our diverse, multidisciplinary team is another strength that facilitated nuanced engagement with the data and the creative use of mixed methods.

## Practice implications and future research

The current findings indicate that it is important for eating disorder treatment across recovery stages to include discussions of social media use. Clearly, use and impacts of social media may differ across diagnoses, and clinicians should be aware of such complexities and nuances. As social media becomes an increasingly important part of everyday life—a key site where individuals access information, communicate with others, and negotiate their identities—social media needs to be understood, for many, as a crucial part of both eating disorders and recovery. The visions of recovery circulating on popular platforms like TikTok likely shape individuals’ expectations of recovery and the ways they orient themselves to treatment and their personal recovery journeys.

Additionally, clinicians should be cautiously curious about the helpfulness of pro-recovery social media during recovery, particularly among individuals seeking recovery from BED given how much diet culture and dieting is being promoted in most-viewed BED recovery spaces on Tik Tok. In spite of the potential benefits of participation in online pro-recovery communities [36], our findings indicate the concomitant circulation of counterproductive content. In particular, BED recovery social media is especially likely to include recovery tips but is also infiltrated by diet culture. Suggestions related to dieting and restriction run counter to treatment for BED, which should promote disruption of restriction given it is a major contributor to the occurrence of binge eating episodes [69, 70]. Finally, clinicians should be open to discussing the impact of restrictive bias in pro-recovery online spaces on any individuals who have loss of control of eating symptoms. These spaces overly represent AN recovery and recoveries related to restriction, not to mention recoveries of White-passing, female-presenting individuals. As such, clinicians should be attuned to the impacts on individuals without these diagnostic presentations or identities who are trying to find pro-recovery representation online.

Regarding future research, more studies should differentiate across diagnostic identification and look at diagnosis-specific hashtags when examining eating disorder related social media. Studies should also move beyond analysis of social media content into understanding the lived experiences of viewers and creators of such content. This would allow for development of prevention and intervention efforts in the eating disorder social media domain based upon a stronger body of mixed-method research. Finally, there is much more to consider regarding how eating disorders are differentially explained and recognized in popular social media platforms, and how different representations of recovery might impact help seeking behaviors and eating disorder recovery more generally.

## Conclusion

Our mixed-methods codebook thematic analysis of 241 TikTok videos provided a nuanced description of the pro-recovery landscape on this popular social media platform and identified variations across five different hashtags that refer to recovery from different eating disorder diagnoses. We believe understanding these differences can provide a better understanding of how individuals who are diagnosed with (or self-identify with) different eating disorder diagnoses see themselves represented on social media and how their recoveries might be differently envisioned. For example, while recovery journeys from AN might often appear to demand inpatient treatment seeking, BED recovery journeys appear more defined by weight loss and caloric restriction. These differential imaginaries likely shape the meanings individuals attribute to their eating disorder, the kind of help they seek, the recovery strategies with which they experiment, their expectations for treatment, and more. We encourage continued investigations into the functionality of pro-recovery on TikTok as well as explorations of how the infiltration of pro-ed content and diet culture promotion into these spaces might impact audience members. Further exploration into how different eating disorders are portrayed on TikTok and other social media platforms is also a rich area of future research.

## Supplementary Information

**Additional file 1.** Themes and Related Codes.

## Data Availability

The datasets used and/or analyzed during the current study are available from the corresponding author on reasonable request.

## Footnotes

1 Notably, we were unable to confirm demographic information of creators. Therefore, we coded for visual characteristics related to gender and race. We acknowledge the limitations of assuming gender and race based upon visual characteristics. We describe these codes as female-presenting and white-passing in order to acknowledge the limitations of our coding. However, it is relevant to code these visual characteristics because these are the same visual data users pull from when making assumptions about creators’ gender and racial identities.

## References

[1] Fardouly J, Vartanian LR (2016). Social media and body image concerns: Current research and future directions. Curr Opin Psychol.

[2] Holland G, Tiggemann M (2016). A systematic review of the impact of the use of social networking sites on body image and disordered eating outcomes. Body Image.

[3] Saiphoo AN, Vahedi Z (2019). A meta-analytic review of the relationship between social media use and body image disturbance. Comput Hum Behav.

[4] Tong ST, Heinemann-Lafave D, Jeon J, Kolodziej-Smith R, Warshay N (2013). The use of pro-ana blogs for online social support. Eat Disord.

[5] Yeshua-Katz D, Martins N (2013). Communicating stigma: the pro-ana paradox. Health Commun.

[6] American Psychiatric Association (2013). Diagnostic and Statistical Manual of Mental Disorders: DSM-5^TM^.

[7] Bratman S, Knight D (2004). Health Food junkies: orthorexia nervosa: overcoming the obsession with healthful eating.

[8] Kang H, Lou C (2022). AI agency versus human agency: understanding human–AI interactions on TikTok and their implications for user engagement. J Comput Mediat Commun.

[9] Gallagher L (2021). Welcome to AnxietyTok: an empirical review of peer support for individuals living with mental illness on social networking site TikTok. Veritas Villanova R J.

[10] Zenone M, Ow N, Barbic S (2021). TikTok and public health: a proposed research agenda. BMJ Glob Health.

[11] Basch CH, Donelle L, Fera J, Jaime C (2022). Deconstructing TikTok videos on mental health: cross-sectional, descriptive content analysis. JMIR Formative Res.

[12] Kong W, Song S, Zhao YC, Zhu Q, Sha L. TikTok as a Health Information Source: Assessment of the Quality of Information in Diabetes-Related Videos. *J Med Internet Res*. 2021;23(9):e30409. 10.2196/30409 10.2196/30409 PMC8444042 34468327

[13] Yeung A, Ng E, Abi-Jaoude E (2022). TikTok and attention-deficit/hyperactivity disorder: a cross-sectional study of social media content quality. Can J Psychiatry.

[14] Greene AK, Maloul EK, Norling HN, Palazzolo LA, Brownstone LM (2023). Systems and selves: an exploratory examination of dissociative identity disorder on TikTok. Qual Psychol.

[15] Giedinghagen A (2023). The tic in TikTok and (where) all systems go: Mass social media induced illness and Munchausen’s by internet as explanatory models for social media associated abnormal illness behavior. Clin Child Psychol Psychiatry.

[16] Müller-Vahl KR, Pisarenko A, Jakubovski E, Fremer C (2022). Stop that! It’s not Tourette’s but a new type of mass sociogenic illness. Brain.

[17] Olvera C, Stebbins GT, Goetz CG, Kompoliti K (2021). TikTok Tics: a pandemic within a pandemic. Mov Disord Clin Pract.

[18] Abi-Jaoude E, Naylor KT, Pignatiello A (2020). Smartphones, social media use and youth mental health. CMAJ.

[19] Primack BA, Shensa A, Escobar-Viera CG (2017). Use of multiple social media platforms and symptoms of depression and anxiety: a nationally-representative study among US Young adults. Comput Hum Behav.

[20] Vidal C, Lhaksampa T, Miller L, Platt R (2020). Social media use and depression in adolescents: a scoping review. Int Rev Psychiatry.

[21] Abbate Daga G, Gramaglia C, Pierò A, Fassino S (2006). Eating disorders and the Internet: cure and curse. Eat Weight Disord.

[22] Tierney S (2006). The dangers and draw of online communication: pro-anorexia websites and their implications for users, practitioners, and researchers. Eat Disord.

[23] Whitehead K (2010). Hunger hurts but starving works: a case study of gendered practices in the online pro-eating-disorder community. Can J Sociol.

[24] Borzekowski DLG, Schenk S, Wilson JL, Peebles R (2010). e-Ana and e-Mia: a content analysis of pro-eating disorder Web sites. Am J Public Health.

[25] Boero N, Pascoe CJ (2012). Pro-anorexia Communities and online interaction: bringing the pro-ana body online. Body Soc.

[26] Harper K, Sperry S, Thompson JK (2008). Viewership of pro-eating disorder websites: association with body image and eating disturbances. Int J Eat Disord.

[27] Peebles R, Wilson JL, Litt IF (2012). Disordered eating in a digital age: eating behaviors, health, and quality of life in users of websites with pro-eating disorder content. J Med Internet Res..

[28] Rodgers RF, Skowron S, Chabrol H (2012). Disordered eating and group membership among members of a pro-anorexic online community. Eur Eat Disord Rev.

[29] Marsh S. TikTok investigating videos promoting starvation and anorexia. The Guardian. https://www.theguardian.com/technology/2020/dec/07/tiktok-investigating-videos-promoting-starvation-and-anorexia. Published December 7, 2020. Accessed June 19, 2023.

[30] TikTok Floods Teens With Eating-Disorder Videos. WSJ. Accessed June 19, 2023. https://www.wsj.com/story/tiktok-floods-teens-with-eating-disorder-videos-b20c2c73

[31] #thyghgapp | Proceedings of the 19th ACM Conference on Computer-Supported Cooperative Work & Social Computing. Accessed June 19, 2023. 10.1145/2818048.2819963

[32] Juarascio AS, Shoaib A, Timko CA (2010). Pro-eating disorder communities on social networking sites: a content analysis. Eat Disord.

[33] Gavin J, Rodham K, Poyer H (2008). The presentation of “Pro-Anorexia” in ONLINE GROUp interactions. Qual Health Res.

[34] Curry J, Ray S (2010). Starving for support: how women with anorexia receive ‘Thinspiration’ on the internet. J Creat Ment Health.

[35] Dias K. The Ana Sanctuary: Women’s Pro-Anorexia Narratives in Cyberspace 2003;4

[36] Au ES, Cosh SM (2022). Social media and eating disorder recovery: an exploration of Instagram recovery community users and their reasons for engagement. Eat Behav.

[37] Branley DB, Covey J (2017). Pro-ana versus pro-recovery: a content analytic comparison of social media users’ communication about eating disorders on Twitter and Tumblr. Front Psychol.

[38] Fettach Y, Benhiba L. Pro-eating disorders and pro-recovery communities on reddit: text and network comparative analyses. In: Proceedings of the 21st international conference on information integration and web-based applications & services. iiWAS2019. Association for Computing Machinery; 2020, pp 277–286. 10.1145/3366030.3366058

[39] Herrick SSC, Hallward L, Duncan LR (2021). “This is just how I cope”: an inductive thematic analysis of eating disorder recovery content created and shared on TikTok using #EDrecovery. Int J Eat Disord.

[40] Riley S, Rodham K, Gavin J (2009). Doing weight: pro-ana and recovery identities in cyberspace. J Commun Appl Soc Psychol.

[41] Harriger JA, Evans JA, Thompson JK, Tylka TL (2022). The dangers of the rabbit hole: Reflections on social media as a portal into a distorted world of edited bodies and eating disorder risk and the role of algorithms. Body Image.

[42] Logrieco G, Marchili MR, Roversi M, Villani A (2021). The paradox of Tik Tok anti-pro-anorexia videos: how social media can promote non-suicidal self-injury and anorexia. Int J Environ Res Public Health.

[43] Mortimer R (2019). Pride Before a fall: shame, diagnostic crossover, and eating disorders. J Bioeth Inq.

[44] Burns M (2004). Eating like an ox: femininity and dualistic constructions of bulimia and anorexia. Fem Psychol.

[45] Saguy AC, Gruys K (2010). Morality and health: news media constructions of overweight and eating disorders. Soc Probl.

[46] Mustelin L, Bulik CM, Kaprio J, Keski-Rahkonen A (2017). Prevalence and correlates of binge eating disorder related features in the community. Appetite.

[47] Hollett KB, Carter JC (2021). Separating binge-eating disorder stigma and weight stigma: a vignette study. Int J Eat Disord.

[48] Santarossa S, Lacasse J, Larocque J, Woodruff SJ (2019). #Orthorexia on Instagram: a descriptive study exploring the online conversation and community using the Netlytic software. Eat Weight Disord.

[49] Valente M, Cesuroglu T, Labrie N, Syurina EV (2022). “When are we going to hold orthorexia to the same standard as anorexia and bulimia?” Exploring the medicalization process of orthorexia nervosa on Twitter. Health Commun.

[50] Lord VM, Reiboldt W, Gonitzke D, Parker E, Peterson C (2018). Experiences of recovery in binge-eating disorder: a qualitative approach using online message boards. Eat Weight Disord.

[51] Padín PF, González-Rodríguez R, Verde-Diego C, Vázquez-Pérez R (2021). Social media and eating disorder psychopathology: a systematic review. Cyberpsychol J Psychosoc Res Cybersp.

[52] Braun V, Clarke V (2021). Can I use TA? Should I use TA? Should I *not* use TA? Comparing reflexive thematic analysis and other pattern-based qualitative analytic approaches. Couns Psychother Res.

[53] Brooks J, McCluskey S, Turley E, King N (2015). The utility of template analysis in qualitative psychology research. Qual Res Psychol.

[54] Cascio MA, Lee E, Vaudrin N, Freedman DA (2019). A team-based approach to open coding: considerations for creating intercoder consensus. Field Methods.

[55] Ghaznavi J, Taylor LD (2015). Bones, body parts, and sex appeal: an analysis of #thinspiration images on popular social media. Body Image.

[56] Wick MR, Harriger JA (2018). A content analysis of thinspiration images and text posts on Tumblr. Body Image.

[57] Jovanovski N, Jaeger T (2022). Demystifying ‘diet culture’: exploring the meaning of diet culture in online ‘anti-diet’ feminist, fat activist, and health professional communities. Women’s Stud Int Forum.

[58] Braun V, Clarke V (2021). To saturate or not to saturate? Questioning data saturation as a useful concept for thematic analysis and sample-size rationales. Qual Res Sport Exerc Health.

[59] Benjamini Y, Hochberg Y (1995). Controlling the false discovery rate: a practical and powerful approach to multiple testing. J Roy Stat Soc Ser B (Methodol).

[60] Sharpe D. Chi-square test is statistically significant: Now what? 10.7275/TBFA-X148

[61] Sonneville KR, Lipson SK (2018). Disparities in eating disorder diagnosis and treatment according to weight status, race/ethnicity, socioeconomic background, and sex among college students. Int J Eat Disord.

[62] Castellini G, Lo Sauro C, Mannucci E (2011). Diagnostic crossover and outcome predictors in eating disorders according to DSM-IV and DSM-V proposed criteria: a 6-year follow-up study. Psychosom Med.

[63] Bulik CM, Sullivan PF, Kendler KS (2000). An empirical study of the classification of eating disorders. Am J Psychiatry.

[64] Matthews KN, Psihogios M, Dettmer E, Steinegger C, Toulany A (2020). “I am the embodiment of an anorexic patient’s worst fear”: severe obesity and binge eating disorder on a restrictive eating disorder ward. Clin Obes.

[65] Duarte C, Pinto-Gouveia J, Ferreira C (2017). Ashamed and fused with body image and eating: binge eating as an avoidance strategy. Clin Psychol Psychother.

[66] Phelan SM, Burgess DJ, Yeazel MW, Hellerstedt WL, Griffin JM, van Ryn M (2015). Impact of weight bias and stigma on quality of care and outcomes for patients with obesity. Obes Rev.

[67] Dunn TM, Bratman S (2016). On orthorexia nervosa: a review of the literature and proposed diagnostic criteria. Eat Behav.

[68] Greene AK, Brownstone LM (2021). “Just a place to keep track of myself”: eating disorders, social media, and the quantified self. Fem Media Stud..

[69] Harvey K, Rosselli F, Wilson GT, DeBar LL, Striegel-Moore RH (2011). Eating patterns in patients with spectrum binge eating disorder. Int J Eat Disord.

[70] Stein RI, Kenardy J, Wiseman CV, Dounchis JZ, Arnow BA, Wilfley DE (2007). What’s driving the binge in binge eating disorder? A prospective examination of precursors and consequences. Int J Eat Disord.

