# Supplementary material for: Visions of recovery: a cross-diagnostic examination of eating disorder pro-recovery communities on TikTok
Source: J Eat Disord. 2023 Jul 3;11:109. doi: 10.1186/s40337-023-00827-7 (PMC10318659; doi:10.1186/s40337-023-00827-7)
Supplement: Supplementary file 1 — Additional file 1. Themes and Related Codes. [file 40337_2023_827_MOESM1_ESM.docx]

**Appendix A: Themes and Related Codes**

|  | Related Codes | Definition |
| --- | --- | --- |
| Centrality of Food to Eating Disorders and Recovery | | |
|  | Food and eating discussed or displayed  Eating/food discussed  Food Visible  Eating Visible | Food and/or eating are discussed in video  Food is shown in video.  Content creator is shown eating or drinking. |
|  | Full day of eating | Video montages or describes all of the food the content creator ate in a day, often accompanied by the hashtags FDOE (Full Day of Eating) or WIEIAD (What I Eat In A Day). |
|  | Fear foods | Content creator describes or consumes something that was previously challenging for them to consume. |
| What Eating Disorders Look and Feel Like | | |
|  | Explaining eating disorders | Provides information about eating disorders or recovery, such as correcting misconceptions or giving definitions. |
|  | Gallows humor | Gallows humor refers to “making fun of a life-threatening, disastrous, or terrifying situation” (Herrick, et al., 2021). |
|  | Personification | Attribution of a personal nature or human characteristics to an eating disorder. |
| Recovery as a Process | | |
|  | Me then and me now | Content creator indicates self-change in the context of recovery through the passage of time. |
|  | Recovery is going well | Content creator shares a subjective success in their recovery. |
|  | Recovery is a struggle | Content creator shares a subjective challenge in their recovery. |
| Giving and Getting Help | | |
|  | Recovery tips | Content creator provides explicit informational advice to help others in the recovery process. |
|  | Showing support | Content creator shares approval, encouragement, or comfort to others in the recovery process. |
|  | Trigger warnings | Video includes explicit verbal or visual cue of “trigger warning” (tw) or “content warning” (cw). |
|  | Treatment | Content creator mentions receiving treatment in any other form besides inpatient (e.g., outpatient therapy, medical providers, etc.). |
|  | Inpatient storytime | Content creator shares everyday experience of  receiving treatment in an inpatient setting (Herrick, et al., 2021). |
| Diet Culture in Recovery | | |
|  | Diet Culture Critique | Challenges societal messaging about dieting, weight loss, overvaluation of thinness “myths about food and eating, and a moral hierarchy of bodies derived from patriarchal, racist, and capitalist forms of domination” (Jovanovski & Jaeger, 2022). |
|  | Diet Culture Promotion | Upholds societal messaging that conflates “weight and health including myths about food and eating, and a moral hierarchy of bodies derived from patriarchal, racist, and capitalist forms of domination” (Jovanovski & Jaeger, 2022). |
